# Efficacy of chemotherapy in elderly patients with unresectable pancreatic cancer: a multicenter review of 895 patients

**DOI:** 10.1186/s12876-017-0623-8

**Published:** 2017-05-22

**Authors:** Taira Kuroda, Teru Kumagi, Tomoyuki Yokota, Nobuaki Azemoto, Aki Hasebe, Hirotaka Seike, Mari Nishiyama, Nobu Inada, Naozumi Shibata, Hideki Miyata, Tomoe Kawamura, Yusuke Imai, Akiko Ueno-Toshimori, Yoshinori Tanaka, Takashi Terao, Yoshiki Imamura, Mitsuhito Koizumi, Hirofumi Yamanishi, Yoshinori Ohno, Yoichi Hiasa, on behalf of the Ehime Pancreato-Cholangiology (EPOCH) Study Group

**Affiliations:** 1 Department of Gastroenterology and Metabology, Ehime University Graduate School of Medicine, To-on, Ehime Japan; 2 Center for Liver-Biliary-Pancreatic Disease, Matsuyama Red Cross Hospital, Matsuyama, Ehime Japan; 3 Department of Gastroenterology, Ehime Prefectural Central Hospital, Matsuyama, Ehime Japan; 4 Department of Internal Medicine, Saiseikai Imabari Hospital, Imabari, Ehime Japan; 5 Department of Gastroenterology, Uwajima Municipal Hospital, Uwajima, Ehime Japan; 6 Department of Gastroenterology, Matsuyama Municipal Hospital, Matsuyama, Ehime Japan; 7 Department of Internal Medicine, Saiseikai Matsuyama Hospital, Matsuyama, Ehime Japan; 8 Department of Gastroenterology, Ehime Prefectural Niihama Hospital, Niihama, Ehime Japan

**Keywords:** Pancreatic cancer, Elderly, Chemotherapy, Best supportive care

## Abstract

**Background:**

The efficacy of chemotherapy for unresectable pancreatic cancer has improved. However, it is occasionally difficult to make treatment decisions for elderly patients. We reviewed the outcomes of elderly patients with unresectable pancreatic cancer by using a large cohort and evaluated whether they had received chemotherapy and the reason why.

**Methods:**

Data for 895 pancreatic cancer patients who were treated using chemotherapy or best supportive care were analyzed considering demographics, clinical stage, treatment, and outcome. Data were analyzed using the chi-square test, Student *t*-test, or Mann-Whitney *U*-test, as appropriate. Outcomes were analyzed using the Kaplan-Meier method. Differences in survival were analyzed using the log-rank test.

**Results:**

The median survival time was significantly shorter in elderly patients (≥65 years) than in younger patients (<65 years) (181 vs. 263 days, *P* = 0.0001). The median survival time of patients treated with chemotherapy was not significantly different between the elderly and the younger group (274 days vs. 333 days, *P* = 0.09), and nor was that of patients choosing best supportive care (84 days vs. 78 days, *P* = 0.83). These results held true even when the age cut-off between younger and elder patients was increased to 70, 75, and 80 years. Elderly patients treated with chemotherapy had a significantly longer median survival time than those choosing best supportive care (274 vs. 86 days, *P* < 0.0001); a significantly greater proportion of elderly patients chose best supportive care compared to younger patients (47.8 vs. 25.8%, *P* < 0.0001). The reason for choosing best supportive care was established in 261 elderly patients (82.9%); 133 (51.0%) met the eligibility criteria for chemotherapy, but of these, 78 (58.6%) were not informed about their disease. The treatment preferences of elderly patients were not always considered; they often received only best supportive care per family members preference (*N* = 65, 48.8%) or because the physician based their treatment decision only on the patient’s age (*N* = 68, 51.1%).

**Conclusions:**

Chemotherapy appears effective for elderly pancreatic cancer patients with unresectable disease, but treatment needs to be optimized to improve prognosis.

## Background

Pancreatic cancer (PC) generally has a very poor prognosis, and is the eighth leading cause of cancer deaths in men and the ninth in women worldwide [1]. In addition, the number of patients with PC is increasing globally [2]. Although surgical resection is the only potentially curative treatment, it is only possible in approximately 10–20% of newly diagnosed patients [1].

Gemcitabine (GEM) or S-1 based chemotherapy has been the primary treatment for patients with unresectable PC in Japan, although recently new regimens have emerged that have given improved outcomes. For example, a combination chemotherapy regimen consisting of oxaliplatin, irinotecan, fluorouracil, and leucovorin (FOLFIRINOX) provided a significant survival advantage compared to GEM [3]. Albumin-bound paclitaxel combined with GEM also improved overall survival in patients with metastatic PC [4]. We previously conducted a retrospective chart review between 2001 and 2010 for 1248 consecutive patients with PC in 10 centers [5]. This revealed that long-term outcomes in PC had improved, suggesting that chemotherapy should be offered to patients previously only considered for best supportive care (BSC).

Many elderly patients with malignancies often choose BSC even though they meet the eligibility criteria for chemotherapy. Under-treatment because of age, regardless of any comorbidity, has been previously reported in patients with malignancies such as lung, colorectal, and breast carcinomas, and lymphoma [6, 7]. The reasons for this bias may include patient preferences, the tendency of physicians to treat patients according to their age, and a lack of evidence-based guidelines for treating elderly patients [6]. Nevertheless, approximately 60% of all new cancer cases and 70% of all cancer-related deaths occur in the elderly (i.e. those aged ≥ 65 years) [8].

The age-specific incidence of PC is also increasing amongst patients aged ≥70 years in Japan, whereas the incidence amongst younger patients remains unchanged [9]. Despite an ageing population, there are relatively few reports on the efficacy and safety of GEM-based chemotherapy for elderly PC patients compared to younger patients [10–13], and those that have been published only included a small number of patients. Here, we report the results of a study conducted by the Ehime Pancreato-Cholangiology (EPOCH) Study Group that used a large patient cohort to examine the efficacy of chemotherapy and the issues faced by elderly patients with unresectable PC.

## Methods

### Study design

This study was a retrospective chart review that included patients who had been diagnosed with PC at Ehime University Hospital or one of its 9 affiliated community hospitals (EPOCH Study Group). The study protocol conformed to the ethical guidelines of the 1975 Declaration of Helsinki, and it was approved by the relevant institutional ethics committee at the Ehime University Graduate School of Medicine (approval number: 1204066). Data were stored in a secure database and patients were numerically coded to anonymize data.

### Patients

We collected the data of consecutive PC patients at 10 centers between January 2001 and December 2010, as previously described [5]. Briefly, PC was diagnosed on the basis of abdominal imaging reported by board certified radiologists, together with histologic findings reported by board certified pathologists if a pathological specimen was retrieved [5]. We included patients who were diagnosed with pancreatic ductal adenocarcinoma, and excluded patients who underwent surgical resection, were diagnosed with a pancreatic neoplasm other than PC, or for whom the relevant data was missing. The final clinical stage was determined on the basis of imaging results. Treatments were divided into 2 categories: chemotherapy (with or without radiation), and BSC. For the purposes of this study, the patients were divided into an elderly group (aged ≥65 years) and a younger group (aged <65 years), as defined by the World Health Organization [14] and the Ministry of Health, Labour and Welfare [15].

### Data collection

Data collected included demographics (age and sex), date of diagnosis, tumor location, clinical stage (JPS TNM classification [16]), treatment, cause of death, and outcome. Some differences in the TNM classification between the JPS and UICC staging systems with respect to tumor factors (T) and lymph node metastases (N) need to be noted. Briefly, T4 in UICC indicates tumor invasion limited to the trunk of the celiac artery or superior mesenteric artery, whereas invasion to any major vessels, neural plexus, or adjacent organs are included in the JPS classification. Positive lymph node metastasis in UICC is designated N1, while in JPS, various grades are used depending on the distance from the main tumor (N1, N2, or N3).

### Analyses of the reason for choosing BSC

We identified the reason for selecting BSC from medical charts to evaluate whether the patients choosing BSC received an optimized treatment. Eligibility criteria for chemotherapy included performance status 0–2, serum aspartate aminotransferase ≤ upper limit of normal (ULN) × 2.5, serum alanine aminotransferase ≤ ULN × 2.5, serum total bilirubin ≤ ULN × 2, serum creatinine ≤ ULN, blood urea nitrogen ≤ ULN, white blood cell count ≥ 4000 /μL, hemoglobin ≥ 9.5 g/dL, platelet count ≥ 10 × 10^4^ /μL, and no complications or dementia that would affect the decision to administer chemotherapy. We defined a non-optimized BSC group as containing patients who met the eligibility criteria for chemotherapy but who chose BSC because (a) they were not informed that they had the disease, (b) the family members preferred this option, or (c) the physician selected this treatment because of the patient’s age.

### Outcome measures

The primary outcome measure was the proportion of patients choosing BSC. The secondary outcome measures included the proportion of patients treated with chemotherapy, the total median survival time (MST), the MST of patients treated with chemotherapy or BSC, and the proportion of patients who choose BSC inappropriately.

### Statistical analyses

All results are expressed as the mean ± standard deviation. Data were analyzed using the chi-square test, Student *t*-test, or Mann-Whitney *U*-test, as appropriate. Outcomes were analyzed using the Kaplan-Meier method. Differences in survival were analyzed using the log-rank test. Two-tailed significance was defined in all analyses as a *P*-value < 0.05. All statistical analyses were performed using the JMP statistical software package (version 11; SAS Institute Japan, Tokyo, Japan).

## Results

### Patients

**Fig. 1:**
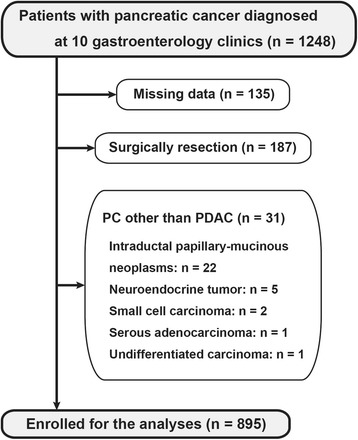
Schematic flow chart of the study design. A total of 895 patients from amongst 1248 patients with unresectable PDAC (71.7%) were included. PC, pancreatic cancer; PDAC, pancreatic ductal adenocarcinoma

A total of 1248 PC patients were enrolled in the study. After an extensive chart review, patients with a final diagnosis of intraductal papillary mucinous neoplasm (*n* = 22), neuroendocrine tumor (*n* = 5), small cell carcinoma (*n* = 2), serous adenocarcinoma (*n* = 1), and undifferentiated carcinoma (*n* = 1) were excluded from the analysis. Patients who underwent surgical resection (*n* = 187) or for whom data was missing (*n* = 135) were also excluded from the analysis. Finally, 895 patients with unresectable PC (659 elderly patients and 236 younger patients) were included and analyzed (Fig. 1).

The demographic features of the two groups are summarized in Table 1. There was an age difference of 20 years between the two groups: the mean age of the elderly patients was 77.2 ± 6.9 years (range, 65–98 years) and that of the younger patients was 57.2 ± 6.1 years (range, 33–64 years). A greater proportion of elderly patients were women (53.7% vs. 34.3%, *P* < 0.0001), but the distribution of clinical stages (Stages I, II, III, IVa, or IVb), tumor locations (including the pancreatic head and other sites), and cause of death (PC related or PC unrelated) were not significantly different between the two groups. Likewise the proportion of patients who were eligible for surgical resection (those with Stage I, II or III disease) did not differ significantly between the elderly (68 patients, 10.3%) and the younger group (22 patients, 9.3%) (*P* = 0.66). A total of 608 patients (67.9%) died during the study period, of whom 584 (96.1%) died of PC.

**Table 1:** Comparison of the baseline demographics of elderly and younger pancreatic cancer patients (*n* = 895)

		Total (*n* = 895)	Elderly group (*n* = 659)	Younger group (*n* = 236)	*P*-value
Age (y)		71.9 ± 11.1	77.2 ± 6.9	57.2 ± 6.1	NA
Sex (Female)		435 (48.6%)	354 (53.7%)	81 (34.3%)	<.0001
Tumor location, including the pancreatic head		506 (56.5%)	376 (57.1%)	130 (55.1%)	0.51
Stage	I	15 (1.7%)	13 (2.0%)	2 (0.8%)	0.25
	II	18 (2.0%)	16 (2.4%)	2 (0.8%)	0.14
	III	57 (6.4%)	39 (5.9%)	18 (7.7%)	0.36
	IVa	232 (25.9%)	172 (26.1%)	60 (25.4%)	0.84
	IVb	573 (64.0%)	419 (63.6%)	154 (65.3%)	0.65
Treatment	CT	519 (58.0%)	344 (43.4%)	175 (60.3%)	<.0001
	BSC	376 (42.0%)	315 (39.8%)	61 (21.0%)	<.0001
Death resulting from PC		584 (96.1%)	419 (95.2%)	165 (98.2%)	0.09

Elderly group: patients aged ≥65 years; Younger group: patients aged <65 years

*Abbreviations*: *CT* chemotherapy, *BSC* best supportive care, *PC* pancreatic cancer, *NA* not applicable

### Proportion of patients treated with chemotherapy or BSC

**Fig. 2:**
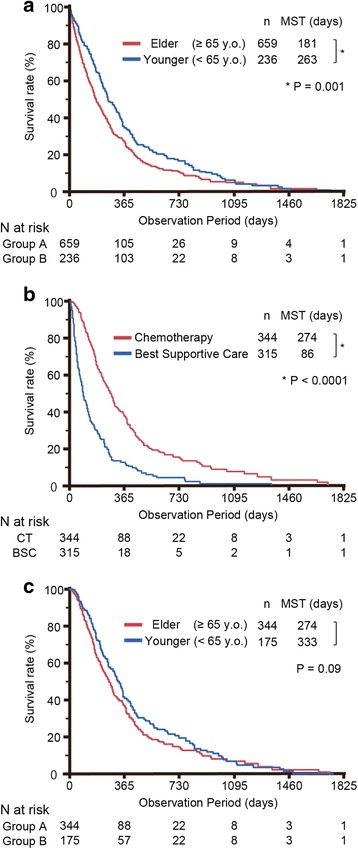
Survival with respect to age and treatment. **a** Elderly group (PC patients aged ≥ 65 years) vs. younger group (PC patients aged <65 years) in all cohorts (*N* = 895). **b** Elderly PC patients who were treated with CT vs. BSC (*N* = 659). **c** Elderly vs. younger PC patients treated using chemotherapy (*N* = 519). MST, median survival time; CT, chemotherapy; BSC best supportive care; PC, pancreatic cancer

Of the 895 patients with unresectable PC, 519 (58.0%) were treated with chemotherapy. The proportion of patients treated with chemotherapy was significantly smaller in the elderly group than that in the younger group (52.2% vs. 74.2%, *P* < 0.001), and correspondingly more of the elderly patients chose BSC (47.8% vs. 25.8%, *P* < 0.001) (Table 1). GEM-based chemotherapy was used in 426 patients (82.1%); GEM + S-1-based, in 44 patients (8.5%); S-1-based, in 36 patients (6.9%); 5-fluorouracil-based, in 9 patients (1.7%); and other agents-based, in 4 patients (0.8%). The demographics of elderly patients in the chemotherapy and BSC groups also varied (Table 2). The BSC group was older (76.5 ± 0.5 years vs. 68.6 ± 0.5 years, *P* < 0.0001), included more female patients (54.3% vs. 44.5%, *P* < 0.0001), and had more patients with tumor involvement in the pancreatic head (63.0% vs. 51.8%, *P* = 0.0013) and earlier stage (Stage I–III) disease (12.5% vs. 8.3%, *P* = 0.0428, Fig. 2).

**Table 2:** Comparison of the baseline demographics of chemotherapy and best supportive care group (*n* = 895)

		Chemotherapy group (*n* = 519)	BSC group (*n* = 376)	*P*-value
Age (y)		68.6 ± 0.5	76.5 ± 0.5	<.0001
Sex (Female)		231 (44.5%)	204 (54.3%)	<.0001
Tumor location, including the pancreatic head		269 (51.8%)	237 (63.0%)	0.0013
Stage	I	4 (0.8%)	11 (2.9%)	0.013
	II	7 (1.3%)	11 (2.9%)	0.10
	III	32 (6.2%)	25 (6.7%)	0.77
	IVa	147 (28.3%)	85 (22.6%)	0.064
	IVb	329 (63.4%)	244 (64.9%)	0.64
Death resulting from PC		364 (98.1%)	220 (92.8%)	0.0011

*Abbreviations*: *BSC* best supportive care, *PC* pancreatic cancer

### Overall survival

The analyses of survival are shown in Fig. 2. The MST was significantly shorter in the elderly group than in the younger group (181 days vs. 263 days, *P* = 0.0001, Fig. 2a). In the elderly group, the MST of patients treated with chemotherapy was significantly longer than the MST of those choosing BSC (274 days vs. 86 days, *P* < 0.0001, Fig. 2b). However, 27 patients (7.8%) who received chemotherapy survived no longer than 90 days. The MST of patients treated with chemotherapy was not significantly different between the elderly and the younger group (274 days vs. 333 days, *P* = 0.09, Fig. 2c), and nor was that of patients choosing BSC (84 days vs. 78 days, *P* = 0.83). These results held true even when the age cut-off between younger and elder patients was increased to 70, 75, and 80 years (data not shown). The BSC group also contained fewer patients who died of PC-related causes compared to the chemotherapy group (98.1% vs. 92.8%, *P* = 0.0011, Fig. 2).

### Analyses of the reason for choosing BSC

Of the 315 patients choosing BSC in the elderly group, it was possible to determine the reason for this choice in 261 patients (82.9%), and 133 of these 261 patients (51.0%) were in fact eligible for chemotherapy. Seventy-eight of these 133 patients (58.6%) were not informed of their diagnosis at the request of their family, 65 (48.8%) were treated based not according to their own preference, but to that of their family, and 68 (51.1%) were treated based only on their age. Of the 61 patients choosing BSC in the younger group, the reason for choosing this could be determined in 35 patients (57.4%). Eleven of these patients (31.4%) met the eligibility criteria for chemotherapy, but 5 (45.5%) were not informed of their diagnosis. The reason for choosing BSC amongst younger patients who met the eligibility criteria for chemotherapy included their own preference in 7 patients (63.6%), family members’ preferences in 2 patients (18.2%), and the physicians’ policy of treating patients according to their age in 2 patients (18.2%).

## Discussion

In our study, GEM-based chemotherapy appeared to be effective for both elderly and younger PC patients. However, we found that a significantly greater proportion of elderly patients chose BSC, which led to a poorer prognosis. In addition, the preferences of elderly patients who were treated with BSC despite being eligible for chemotherapy were not taken into account, mainly because their family had asked that they not be informed of their diagnosis.

In elderly patients, the use of chemotherapy is often limited due to the increased risk of adverse events related to comorbidities, poor performance status, or poor cognitive function [17]. However, some elderly patients are eligible for chemotherapy, and each case needs to be considered on its own merit. It has been reported that elderly cancer patients are still under-represented in new cancer treatment clinical trials [18–21], and only a few studies have provided information on the tolerability and benefits of chemotherapy in the elderly [22–24]. Consequently, the safety and efficacy of new chemotherapy regimens for PC such as FOLFIRINOX or combined albumin-bound paclitaxel and GEM remains unclear in elderly patients [3, 4]. On the other hand, the safety and efficacy of conventional chemotherapy for PC, mainly GEM-based regimens, has been proven in a number of studies with small patient cohorts, as described above [10–13]. In fact, in the large cohort study we report here, GEM-based chemotherapy was equally effective in younger and elderly patients. This is consistent with the results of previous studies [10–13], and suggests that GEM-based chemotherapy may continue to play a more important role than novel regimens in elderly patients until the safety and efficacy of the latter are proven.

Our data also showed that quite a large number of elderly patients chose BSC even though they met the eligibility criteria for chemotherapy, indicating that simply providing the best treatment option may improve the prognosis of these patients. A previous study showed that many patients receiving chemotherapy for incurable cancers might not understand that chemotherapy is unlikely to be curative [25]. On the other hand, there may be a number of reasons why elderly patients tend to choose BSC over chemotherapy, which are likely to include organ dysfunction, drug toxicity, and a refusal to give informed consent. Yamagishi et al. suggested that these include patient disappointment that chemotherapy does not cure unresectable cancer, the greater susceptibility of elderly patients to severe drug toxicity that is most likely due to the age-related deterioration of organ function and/or drug clearance, and other disorders that may contraindicate chemotherapy [10]. A particular point of concern is the high prevalence of patients who were not informed about their medical condition based on a family member’s wishes and who then chose BSC (41.1%). Although the informed consent rate at a cancer institute was as high as 75% in 1997, data from the Ministry of Health, Labor and Welfare in Japan shows that the informed consent rate among cancer patients in general practice has been gradually increasing but is still not acceptably high: 18% in 1992, 20% in 1994, 50% in 1998, 66% in 2006, and 74% in 2012. These studies included all cancer patients, but when the analysis was limited to patients who were predicted to survive less than 6 months, the informed consent rate was only 46% in 2005.

In order to better understand and improve inaccurate beliefs about chemotherapy, sufficient recognition of its limitations in elderly patients is needed among medical staff and the patients’ family. Care should be taken to avoid misleading the patient due to insufficient knowledge and a lack of evidence-based informed consent, which in turn would allow the patients’ preferences to be better reflected. Although the cut-off age of 65 years might not match the current clinical view of what “elderly” means, our findings held true even when the age cut-off between elderly and non-elderly patients was increased to 70, 75, or even 80 years.

Our study has several limitations. First, not all patients had histologically proven adenocarcinoma because fine needle aspiration was not widely used in general practice, especially in the first half of the study period and this retrospective study was conducted mainly among community hospitals where fine needle aspiration is not yet available. Nevertheless, given that the vast majority (87%) of Japanese PC patients who were assessed histologically were diagnosed with adenocarcinoma [26], this may have had only a small impact on the treatment strategy and prognosis in the current study population. Second, the reason for choosing BSC was not identified in 54 elderly patients (17.1%) and this may overestimate the reason why they chose BSC. However, the high proportion (78 of 133, 58.6%) of cases in which BSC may have been selected inappropriately by elderly patients is the more important issue. Third, our study mainly included patients treated with GEM-based chemotherapy, rather than novel regimens, because these new treatments had not been approved within this study period in Japan. Further clinical trials are required to address adaptations of these novel regimens for elderly PC patients. We also need to assess drug toxicity, which was not possible in this study. Finally, some patients who are eligible for chemotherapy have an unfavorable prognosis and do not benefit from it. Indeed, in our study, 27 elderly patients (7.8%) who received chemotherapy survived no longer than 90 days. Furthermore, eligibility criteria for chemotherapy other than age, performance status, liver function, and renal function may exist. It is therefore important to identify predictive factors for whether chemotherapy will benefit elderly patients.

## Conclusion

Chemotherapy appears to be well tolerated and effective among elderly patients with PC. However, a considerable number of patients who might have benefited from chemotherapy instead chose BSC. A strategy to provide the best possible treatment option for each patient with unresectable PC is needed in order to improve the prognosis, especially in elderly patients.

## Abbreviations

BSC: Best supportive care
FOLFIRINOX: A combination chemotherapy regimen consisting of oxaliplatin, irinotecan, fluorouracil, and leucovorin
GEM: Gemcitabine
JPS: Japan Pancreas Society
MST: Median survival time
PC: Pancreatic cancer
ULN: Upper limit of normal
